# Tuning Collective Plasmon Resonances of Femtosecond Laser-Printed Metasurface

**DOI:** 10.3390/ma15051834

**Published:** 2022-03-01

**Authors:** Dmitrii Pavlov, Alexey Zhizhchenko, Lei Pan, Aleksandr A. Kuchmizhak

**Affiliations:** 1 Institute of Automation and Control Processes, Far Eastern Branch, Russian Academy of Science, 5 Radio Str., 690041 Vladivostok, Russia; pavlov_dim@mail.ru (D.P.); g89leksig@mail.ru (A.Z.); 2 Far Eastern Federal University, 690041 Vladivostok, Russia; 3 MIIT Key Laboratory of Critical Materials Technology for New Energy Conversion and Storage, School of Chemistry and Chemical Engineering, Harbin Institute of Technology, Harbin 150001, China; panlei@hit.edu.cn; 4 Pacific Quantum Center, Far Eastern Federal University, 690041 Vladivostok, Russia

**Keywords:** femtosecond laser pulses, noble metal films, direct laser processing, plasmonic nanostructures, collective resonances

## Abstract

The optical response of properly excited periodically arranged plasmonic nanostructures is known to demonstrate sharp resonance features associated with high-Q collective modes demanding for various applications in light–matter interaction, filtering and sensing. Meanwhile, practical realization and replication of plasmonic platforms supporting high-Q modes via scalable inexpensive lithography-free approach is still challenging. Here, we justify direct ablation-free irradiation of Si-supported thin Au film by nanojoule-energy femtosecond laser pulses as a single-step and scalable technology for realization of plasmonic metasurfaces supporting collective plasmonic response. Using an adjustable aperture to control and upscale the size of the fabricated nanostructures, nanobumps and nanojets, we demonstrated plasmonic metasurface supporting collective resonances with a moderately high Q-factor (up to 17) and amplitude (up to 45%) within expanded spectral range (1.4–4.5 µm). Vacuum deposition of thin films above the as-fabricated nanostructure arrays was demonstrated to provide fine tuning of the resonance position, also expanding the choice of available materials for realization of plasmonic designs with extended functionality.

## 1. Introduction

Coupling of the propagating electromagnetic waves into resonant oscillations of free electron plasma (surface plasmons (SPs)) propagating at the interface of the noble metal and dielectric is widely used to enhance light–matter interaction in novel optoelectronic devices, metasurfaces and chemo- and biosensors [1,2,3,4,5,6]. SPs can be localized within nanoscale noble metal structures acting as the resonators, while their geometry defines the spectral position and Q-factor of the SP resonance. Meanwhile, high intrinsic losses inherent to commonly used noble metals typically limit Q-factor of the related SP nanostructures. However, properly arranged isolated nanostructures can exhibit collective plasmonic resonances (CPRs), associated with coupling between the localized SPs and either diffracted waves or propagating SPs, allowing the excitation of specific modes exhibiting a high Q-factor of the resonance even with lossy metals [7,8,9,10]. Observation of such high-Q modes typically requires either an angled excitation of the nanostructure array or index matching liquid to compensate the difference between the refractive indices at both interfaces of the SP and the active material. Strong angular dispersion of the CPRs also complicates their observation with a pump radiation focused by an objective with moderate and high numerical apertures (NA > 0.3). The need to precisely control both the geometry and arrangement of the nanostructures in the array also requires the involvement of rather time- and money-consuming lithography-based fabrication technologies such as electron- or ion-beam milling and photolithography. The mentioned technologies are typically multistep and nonscalable, limiting their applicability for fabrication of the real devices for routine tasks. In this respect, practical realization of inexpensive plasmon platforms supporting CPR is still challenging.

Among others, laser-based technologies have been rapidly evolving during recent decades owing to appearance of the rather stable and inexpensive sources of ultrashort femtosecond (fs) laser pulses [11]. Ultrafast deposition of the temporally and laterally confined laser energy to the material allows shrinking the heat-affected zone to the lateral scale comparable to the optical diffraction limit making the technology suitable for nanofabrication [12]. Moreover, femtosecond laser excitation of the materials can launch different physical processes (ranging from lattice thermalization and phase transitions to acoustic waves and stress relaxation [13,14,15,16]) that affect the resulting surface morphology, opening up pathways for control and optimization. Noteworthily, single-step chemical-free laser processing is beneficial for the fabrication of plasmonic nanostructure arrays supporting collective plasmonic effects. Direct interaction of the femtosecond laser pulse with a thin substrate-supported noble-metal film was shown to produce unique 3D surface morphologies, nanobumps and nanojets, upon ultrafast laser-induced melting of the metal film, subsequent acoustic relaxation of the molten part from the substrate, liquid-phase reshaping and resolidification [17,18,19,20,21,22,23]. Being arranged into the periodic array at micrometer spacing, the mentioned nanostructures demonstrate resonant response in the near- and mid-IR spectral ranges [24]. Earlier work [25] misinterpreted the resonant IR response of the laser-fabricated nanobumps (nanojets) as a result of geometry-dependent localized plasmon resonances of the isolated nanostructures. Meanwhile, the subsequent works revealed the key role of the CPRs in the response of the nanobump (nanojet) arrays observed in the near- and mid-IR spectral ranges [26]. Such versatile easy-to-fabricate plasmonic metasurfaces were proved to be useful for biosensing [26,27,28], boosting nonlinear effects [29] and empowering the light-emitting properties of an attached layer composed of IR quantum dots [30].

Uniquely, the CPR position of such nanostructure arrays can be controlled by both the geometry of the nanobumps and the geometric spacing between them as both parameters define the “effective” array period [31]. The former can be tailored to some extent by applied pulse energy *E*. However, for the fixed laser focusing conditions (tight focusing was used so far) the nanobump geometry can be tailored only within a certain range of *E* until the formation of the through hole in the metal film at elevating applied pulse energy. Once the maximal nanobump (nanojet) size is fixed by laser processing parameters, the “effective” array period can be increased by moving apart the adjacent nanostructures (increase in the real geometric spacing between the nanobumps), allowing the scalable redshift of the spectral position of the collective plasmon resonance. However, the resonance amplitude gradually decreases owing to weakening coupling between the neighboring nanobumps. The mentioned features reduce the tuning range of the CPR supported by the nanobump array, limiting the performance and practical applicability of such a plasmonic platform.

Here, we addressed these issues, showing that the geometry of the laser-fabricated nanobumps (nanojets) can be simply upscaled by expanding the lateral size of the laser beam through the effective numerical aperture of the focusing lens. On-demand tunability of the nanobump geometry provides the way for single-step direct laser fabrication of the nanostructure arrays supporting CPRs with a high Q-factor (up to 17) and resonance amplitude (up to 45%) within expanded spectral range (1.4–4.5 µm). Vacuum deposition of thin films above the fabricated nanobump arrays provides an additional way for fine tuning the CPR position, also allowing the creation of advanced plasmonic designs with extended functionality.

## 2. Materials and Methods

**Figure 1:**
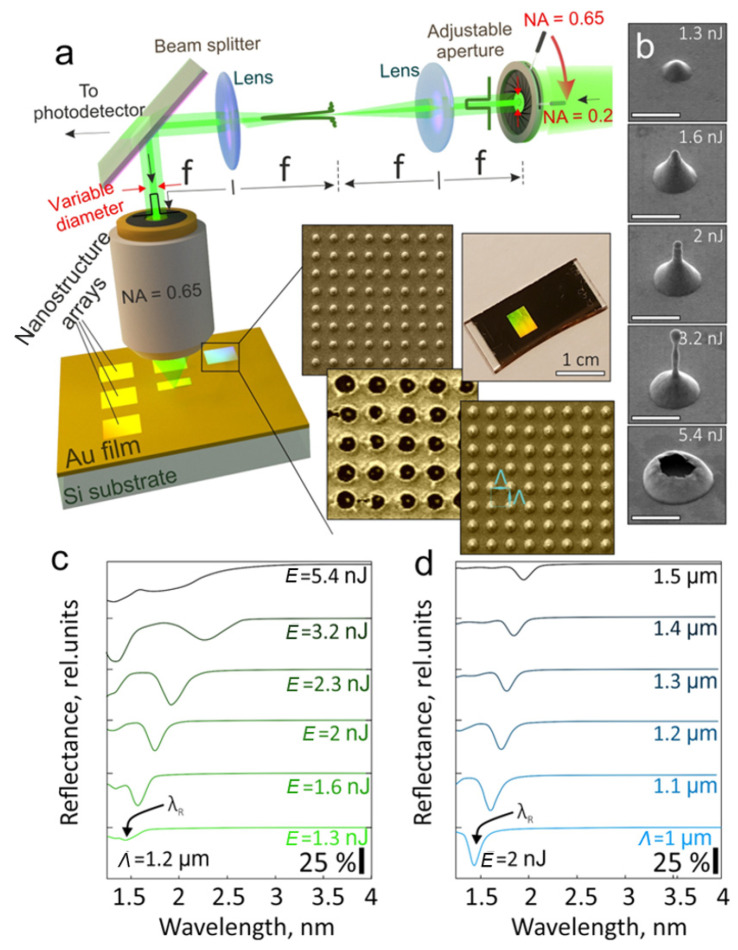
(**a**) Schematically illustrated setup for direct laser fabrication of nanostructure arrays using adjustable aperture to control the effective NA of the focusing objective. Insets show SEM images of typical nanostructure arrays with square arrangement and a periodicity *Λ* as well as optical photograph of the large-scale nanobump array. (**b**) Series of side-view SEM images of the isolated nanostructures produced at NA = 0.65 and applied pulse energy *E* ranging from 1.3 to 5.4 nJ. (**c**,**d**) FTIR reflectance spectra of the nanostructure arrays produced at variable pulse energy *E* and fixed array period *Λ* = 1.2 µm (**c**) and variable *Λ* and fixed *E* = 2 nJ (**d**).

Nanostructure arrays were produced on the surface of a 50 nm thick Au film. The films were directly coated (without any adhesive sublayers) onto monocrystalline silicon (Si) wafers using electron-beam deposition. The same procedure was applied for deposition of additional layers (Al_2_O_3_ and Pt) onto the produced nanostructure arrays. Nanobump (nanojet) arrays were fabricated using direct spot-by-spot irradiation of the Au film surface by 230 fs second-harmonic (central wavelength *λ* of 515 nm) laser pulses coming from a regenerative amplified Yb:KGW-based femtosecond laser system (Light Conversion Ltd., Pharos, Vilnius, Lithuania). The laser beam was focused onto the film surface by a dry microscope objective with a numerical aperture NA = 0.65 yielding a maximal optical spot size of *D_opt_* ≈ 1.22*λ*/NA = 0.96 µm. To reduce the effective NA of the objective (increase *D_opt_*), the calibrated aperture together with a 4*f* optical system was introduced into the optical path of the laser beam to adjust the diameter of the laser beam with respect to the entrance pupil of the microscope objective (see Figure 1a).

Each resulting array contained an identical number (100 × 100) of nanostructures arranged in a square lattice with a periodicity *Λ*. To do this, laser processing was performed by fixing the Au-coated Si wafers on a high-precision PC-driven nanopositioning platform (Aerotech GmbH, ANT 130XY and ANT 130LZS, Nurnberg, Germany) synchronized with the femtosecond laser system. For comparison, we also produced disordered nanostructure arrays produced without synchronization (i.e., by simply scanning the film surface at fixed speed and pulse repetition rate along a snake-like trajectory).

Surface morphology of the nanobumps was studied by a scanning electron microscope (SEM; Carl Zeiss, Ultra 55+, Oberkochen, Germany). Resonant optical properties of the nanostructure arrays in the near- and mid-IR part of the spectrum were evaluated using a Fourier transform infrared (FTIR) spectrometer (Bruker, Vertex 80v, Billerica, MA, USA) coupled to an IR microscope (Bruker, Hyperion 2000). The FTIR reflection signals from the nanostructure arrays were acquired under normal incidence using an incoherent light source focused by a reflective objective with a NA = 0.4. The reference reflection was taken from a bulk Ag mirror. The signal acquisition area was adjusted by a built-in knife-edge aperture to fit the different lateral sizes of the nanostructure arrays produced at variable *Λ*.

## 3. Results and Discussion

The series of side-view SEM images in Figure 1b illustrates how the morphology of the nanostructure produced under single-pulse irradiation of the Si-supported Au film evolves upon the increase in the applied pulse energy *E* in the range 1.3 to 5.4 nJ that corresponds to applied laser fluence ranging from ≈0.19 to 0.8 J/cm^2^. As can be seen, at elevating pulse energy the nanostructure’s geometry changes from the parabola-shaped nanobump to the upright-standing nanojet that finally collapses and forms a micron-diameter through hole in the metal film. A representative series of FTIR reflection spectra of the corresponding nanostructure arrays produced in a square arrangement with a periodicity *Λ* = 1.2 µm is shown in Figure 1c. A typical view of the nanostructure arrays is given by the SEM images on the inset of Figure 1a. As can be seen, when the nanostructures reach a certain size, the dip at a resonant wavelength *λ_R_* appears in the reflection spectrum. The origin of the resonant dip was previously justified to be caused by destructive interference of the surface plasmons excited on the curvy nanostructure’s surface [24]. Upright-standing geometry of the nanojets allows efficient coupling of the normally incident electromagnetic waves to the SPs, which are excited simultaneously on each nanostructure in the array and then travel along the curvy nanobumps and smooth Au film area. Noteworthily, such efficient coupling to the SPs is impossible on a smooth Au film surface owing to the mismatch of the wave vectors of the incident and the surface waves. Consequently, further increase in the nanostructure size causes the redshift of the CPR position *λ_R_* ∝ *Λ_eff_* as the “effective” period is defined not only by the geometric periodicity *Λ* of the array but also by the length of the curvy part (that changes with the nanostructure geometry). In addition, both the Q-factor of the resonance (a ratio of the full width at half maximum *Δ* and *λ_R_*) and the amplitude of the resonance decrease for the arrays fabricated from the elongated nanojets. This is caused by uncontrollable variation of the geometry from one structure to another in the array due to the development of the Rayleigh–Plateau hydrodynamic instability in the elongated part of the liquid-phase nanojet until its resolidification [19,32]. Finally, the array of through microholes formed at pulse energy *E* > 5.4 nJ demonstrates a low-intensity and broad dip in the reflection spectrum. The spectral position of this dip is defined by the geometrical period of the array *Λ* magnified onto the effective refractive index of the surface plasmon wave supported by the metal–dielectric interface [33,34]. Detailed analysis of the resonant optical properties of the laser-fabricated hole arrays is beyond the scope of this study. The interested reader is referred to the recent papers discussing laser fabrication and IR optical properties of such arrays [35,36,37].

The presented FTIR spectra highlight a rather narrow range of highly reproducible nanostructure geometries (defined by applied pulse energy/fluence) that allows fabricating an array exhibiting intense and high-Q CPRs. An increase in the array periodicity *Λ* provides the possibility to expand the CPR tuning range. However, the CPR amplitude gradually decreases owing to weakening coupling between the neighboring nanostructures (the *λ_R_* increases with respect to the nanostructure’s size), as is illustrated by a series of FTIR reflection spectra in Figure 1d. In particular, the CPR amplitude is nearly halved when the array periodicity *Λ* increases from 1 to 1.5 µm.

**Figure 2:**
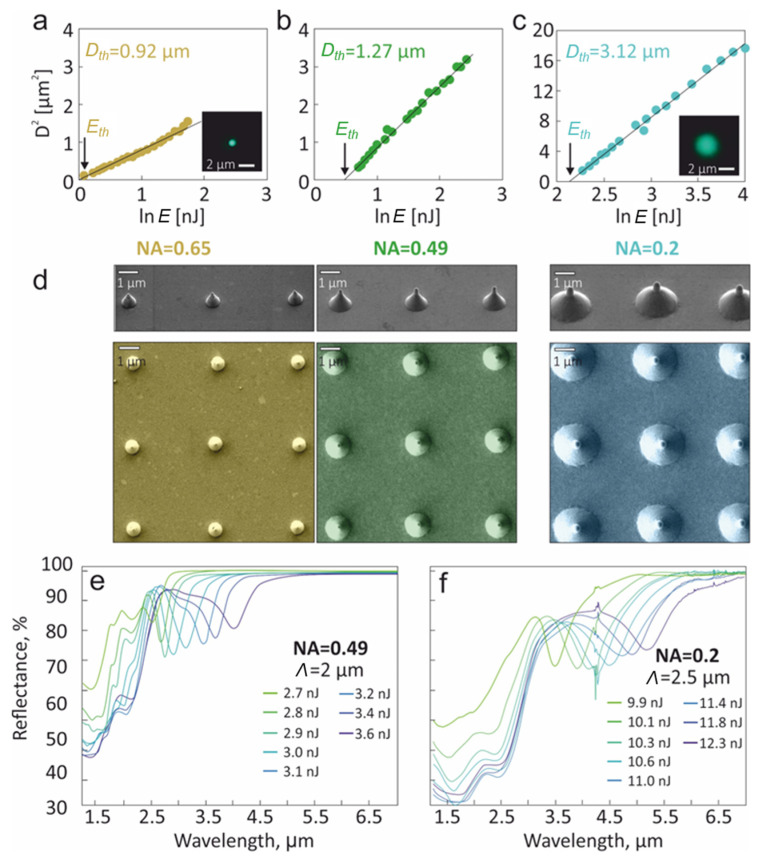
(**a**–**c**) Squared lateral size of the surface modification (nanobump or nanojet) *D*^2^ as a function of the natural logarithm of the applied pulse energy ln*E* (in nJ) measured for three calibrated positions of the iris aperture that tailors the optical diameter *D_opt_* of the laser focal spot. Corresponding linear fits of the obtained data give the characteristic energy deposition diameters *D_th_* of 0.92 (**a**), 1.27 (**b**) and 3.12 µm (**c**). The intersection of the linear fit with the *x*-axis indicates the threshold applied pulse energy *E_th_* = 1 (**a**), 1.7 (**b**) and 8.4 nJ (**c**). Insets in (**a**,**c**) provide focal-plane optical images of the resulting laser beam used for nanostructure fabrication. (**d**) Series of side-view (view angle of 45°) and top-view SEM images showing a scalable increase in the geometric dimensions of the laser-fabricated nanostructures (a nanobump with a small nanojet atop) upon the decrease in the effective NA of the microscope objective from 0.65 to 0.2 using an adjustable iris aperture. All nanostructures were produced at a fixed fluence of 0.21 ± 0.02 J/cm^2^. (**e**,**f**) FTIR reflectance spectra of the nanostructure arrays (200 × 200 nanostructures) produced using laser beam focusing at effective NA of 0.49 (**e**) and 0.2 (**f**). The array period *Λ* was 2 (**e**) and 2.5 µm (**f**).

Analysis of the presented data indicates that translation of the high-Q CPR to practically relevant mid-IR spectral range requires a scalable increase in the nanostructure size that can not be achieved only by increasing *E* (as the nanobump finally collapses, forming a through hole; Figure 1b). To upscale the size of the nanobumps, we introduced an adjustable aperture that reduces the diameter of the laser beam incident onto the entrance pupil of the focusing objective (see Section 2 and Figure 1a). This allowed the gradual decrease in the effective NA of the objective, resulting in the increase in the focal spot size *D_opt_* ≈ 1.22*λ*/NA. Such an approach allowed the flexible tuning of the focal spot size without changing other properties of the optical system used for laser processing. To calibrate the several positions of the aperture regarding the obtained effective NA, we systematically measured the lateral size of the surface modification (either nanobump or nanojet) *D* versus the applied pulse energy *E*. Plotting the obtained dependencies as *D*^2^(ln*E*), one can estimate the characteristic energy deposition diameter *D_th_* according to the slope of the linear fit of these dependencies (Figure 2a–c) [38]. Noteworthily, *D_th_* is generally defined by the initial optical diameter of the laser focal spot *D_opt_*, also considering the constant contribution of the thermal spreading of the molten front in the material ∝ *χ*·*τ* (where *χ* is a thermal diffusivity of Au, while *τ* is characteristic lattice thermalization time required for solid-to-liquid transition). The contribution of the thermal spreading term to the *D_th_* value was shown to be weak at near-threshold pulse energies that correspond to the processing regime used for the nanobump and nanojet formation [22], making *D_th_* ≈ *D_opt_*.

In the case where the aperture is completely open (Figure 2a), the *D_th_* value calculated from the corresponding *D*^2^(ln*E*) dependence was found to be ≈ 0.92 µm, giving the effective numerical aperture of NA ≈ 1.22*λ*/*D_th_* = 0.66 that is in a reasonable agreement with the tabulated value for the used microscope objective. Two additional examples of the measurements carried out for calibrated positions of the adjustable aperture give the *D_th_* values of 1.27 and 3.12 µm, yielding corresponding values of the effective NA of 0.49 and 0.2, respectively (Figure 2b,c). The obtained values of *D_th_* are in reasonable agreement with the visible focal-plane size of the laser beam (see insets in Figure 2a,c). Noteworthily, the threshold fluence *F_th_* = 4·*E_th_*/*πD*^2^*_th_* (where *E_th_* is a threshold pulse energy defined by the intersection of the linear fit of *D*^2^(ln*E*) with the *x*-axis [38]) required for the formation of the nanobump was found to be ≈0.12 ± 0.015 J/cm^2^ for all the presented calibrated positions of the aperture. The obtained *F_th_* value is in agreement with the previously reported data [22], also confirming the reliability of the performed NA calibration procedure. More importantly, by systematically decreasing the effective NA of the focusing objective, we achieved a scalable increase in the lateral and vertical dimensions of the laser-fabricated nanobumps and nanojets, as is revealed by the series of side-view SEM images of the nanostructures produced at constant fluence (Figure 2d). In its turn, this allowed the redshift of the CPR spectral position *λ_R_* for the nanostructure arrays composed of such upscaled nanobumps and nanojets preserving the rather large resonance amplitude up to 35%. To illustrate this, Figure 2d,e provides the series of FTIR spectra of the nanostructure arrays produced at NA = 0.49 (*Λ* = 2 µm) and NA = 0.2 (*Λ* = 2.5 µm) upon the increase in the applied pulse energy *E*.

**Figure 3:**
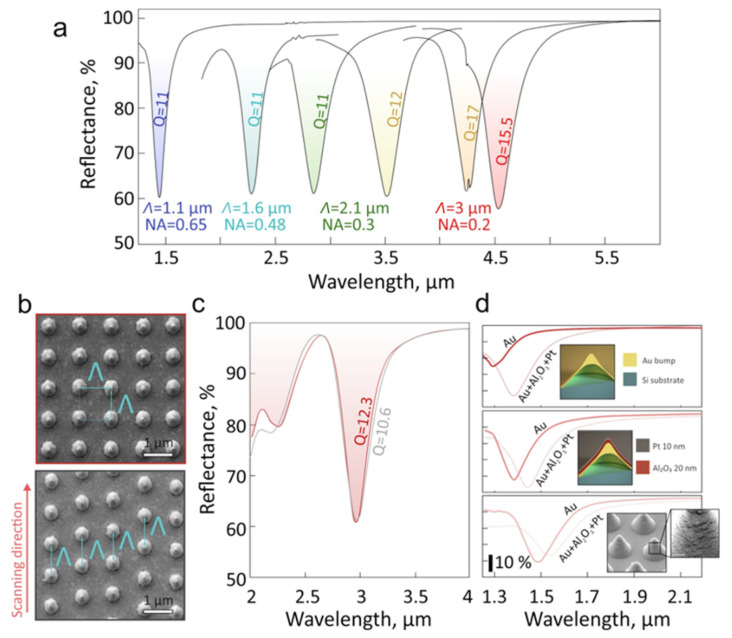
(**a**) Series of representative FTIR reflection spectra of the nanobump arrays exhibiting the highest CPR Q-factor for the different combinations of *Λ*, *E* and effective NA of the focusing objective. All nanostructure arrays were produced by gradually increasing laser fluence from 0.213 to 0.265 J/cm^2^ from left to right. (**b**) Top-view SEM images of the nanobump arrays (*Λ* = 2 µm and *E* = 4.5 nJ) fabricated in square and disordered arrangements and their corresponding FTIR reflectance spectra. (**c**) FTIR reflection spectra of the nanobump arrays produced with (top) and without (bottom) synchronization of the femtosecond laser system with the nanopositioning stages. Both arrays were printed at fluence of 0.24 J/cm^2^ and NA = 0.3. (**d**) Series of FTIR reflectance spectra of the nanostructure arrays before (solid curves) and after their consecutive capping with a 20 nm thick Al_2_O_3_ layer and a 10 nm thick Pt layer (dashed curves). The nanostructure arrays were produced at *Λ* = 1 µm and applied pulse energy *E* = 1.6 (top), 2.5 (middle) and 2.9 nJ (bottom) using NA = 0.65. Top inset schemes compare the nanobump geometry, while the bottom inset SEM images illustrate the nanoscale morphology of the coated nanobump. Scale bar is 100 nm.

The presented results clearly demonstrate that for the fixed focusing conditions there is an optimal combination of the geometry of the laser-fabricated nanostructures and spacing between them providing optimal plasmonic performance (highest amplitude and CPR Q-factor). By systematically adjusting the effective NA of the objective (from 0.65 to 0.2) and varying *E* (fluence) and *Λ*, we fabricated nanobump arrays exhibiting competitive performance (CPR Q-factor larger than 10 and amplitude of 40%) within a rather broad spectral range spanning from 1.4 to 4.5 µm (Figure 3a). The highest Q-factor, 17 (*λ_R_* = 4.23 µm), was achieved for the smallest tested effective numerical aperture, NA = 0.2. This indicates the best reproducibility of the array period and nanostructure shape (in particular owing to larger Rayleigh length of the laser focal spot and weaker effect of the short-term pulse-to-pulse laser stability on the nanostructure geometry). The mentioned performance is comparable with those recently reported for mid-IR plasmonic metasurfaces supporting quasi bound states in the continuum under their excitation with a polarized light [39,40]. The experimentally demonstrated plasmonic performance of the nanobump arrays is almost polarization-insensitive, as was confirmed by measuring the FTIR reflection spectra under unpolarized and linearly polarized excitation of the nanostructure arrays (not shown here). In addition, the demonstrated performance of the laser-fabricated metasurfaces is slightly inferior to those achieved for common nanoparticle arrays that require an application of index-matching layers, oblique excitation and nonscalable multi-step fabrication [10,41,42].

To produce the nanobumps arranged within an ordered lattice, synchronization of the femtosecond laser system with the nanopositioning stages is required. This complicates the experimental setup, also increasing the overall fabrication time of the entire nanostructure array. By scanning the sample surface at a constant speed with laser pulses coming at a constant repetition rate, similar nanostructure arrays with slightly disordered lattices can be produced without any synchronization (Figure 3b). The fabricated array has a constant period *Λ* between neighboring nanostructures only within a certain chosen scanning direction (along the *y*-axis in our case) and random lateral shift between the subsequently fabricated vertical rows. Remarkably, the resulting disordered nanostructure array demonstrated only a slightly lower Q-factor of 10.6 compared to the value obtained for a similar array composed of the same nanostructures arranged into a square lattice with a Q-factor = 12.3 (Figure 3c).

Finally, a postprocessing procedure can be also applied to precisely tune the plasmonic properties of the as-fabricated nanostructure arrays as well as to expand the device functionality. To illustrate this modality, using electron-beam sputtering we consequently coated the nanobump arrays with a 20 nm thick Al_2_O_3_ layer and 10 nm thick Pt layer. FTIR reflectance spectra in Figure 3d show that application of additional layers does not strongly deteriorate the CPR-mediated plasmonic response of the nanostructure arrays produced at fixed *Λ* = 1 µm and variable *E*. Capping nanolayers in this case can be considered as an effective medium modifying the refractive index of the surface plasmon wave supported by the metal film interface that results in the evident redshift of the CPR spectral position [26]. For certain nanostructures, the CPR amplitude increases, showing that an optimal combination of the nanostructure geometry and arrangement can be achieved for the as-fabricated array via the deposition of an additional layer. Such a layer can be used to provide extra functionality important for device realization. For example, the laser-fabricated nanostructure arrays can be coated with a thin layer of chemically active material (Pd, metal oxides, etc.), opening pathways for the realization of plasmonic molecular/gas sensors based on spectral detection of the CPR signal variation [43,44] or surface-enhanced IR absorption (SEIRA) effect [45]. Noteworthily, SEIRA-based sensing requires spectral matching of a certain molecular vibration band with a CPR position that was addressed in this paper by adjusting laser fabrication conditions (*E*, *Λ* and *D_opt_*). This allows fabricating multipurpose (SERS, SEIRA, refractive index) sensor elements with pixelated design [46], where each pixel will contain nanostructure arrays with a certain CPR spectral position. Finally, although the demonstrated nanostructures preserve in-plane circular symmetry, the laser beam shaping can be adopted for the fabrication of asymmetrical elliptical nanobump/nanojets to achieve a polarization-sensitive optical response. The realization of pixelated plasmonic sensors based on laser-fabricated symmetrical and asymmetrical nanobump arrays will become a subject of our forthcoming studies.

## 4. Conclusions

To conclude, the presented results justify facile and scalable direct femtosecond laser patterning as a promising chemical-free method for fabrication and replication of high-Q plasmonic metasurfaces for light–matter interaction, sensing, nonlinear optics and optoelectronics. The demonstrated nanostructure arrays exhibit polarization-insensitive collective plasmonic response with an amplitude of up to 45% and a resonance Q-factor of up to 17. The spectral position of the CPR was tuned between 1.4 and 4.5 µm by adjusting laser fabrication conditions and nanostructure spacing. Fabrication of the nanobumps can be realized on the surface of any thin noble (semi-noble) metal film covering glass, semiconductor or polymer substrates, expanding potential designs and application range. Further translation of the CPR toward practically relevant mid-IR spectral range can be evidently achieved by further upscaling the size of the nanobumps and nanojets. Our results pave the way towards facile fabrication of multipurpose application-ready plasmonic metasurfaces with adjustable polarization-insensitive optical properties where the laser beam shaping [24] together with multibeam interference approaches can be readily adopted to ensure economically justified fabrication rate and resulting device price [47,48,49]. For example, compared to another common single-step fabrication procedure, FIB milling, the suggested approach can be easily scaled up to cover square centimeter surface areas by combining megahertz repetition rate lasers, fast laser beam scanners and adjustable beam shapers.

## Data Availability

Samples are available from the authors.
